# Intratesticular versus intraperitoneal injection of Busulfan for the induction of azoospermia in a rat model

**DOI:** 10.1186/s40360-022-00587-1

**Published:** 2022-07-14

**Authors:** Halimeh Mobarak, Reza Rahbarghazi, Mohammad Nouri, Mohammad Heidarpour, Mahdi Mahdipour

**Affiliations:** 1 Department of Clinical Sciences, Faculty of Veterinary Medicine, Ferdowsi University of Mashhad, Mashhad, Iran; 2 Stem Cell Research Center, Tabriz University of Medical Sciences, Tabriz, Iran; 3 Department of Applied Cell Sciences, Faculty of Advanced Medical Sciences, Tabriz University of Medical Sciences, Tabriz, Iran; 4 Department of Reproductive Biology, Faculty of Advanced Medical Sciences, Tabriz University of Medical Sciences, Tabriz, Iran

**Keywords:** Azoospermia, Animal model, Busulfan, Toxicity

## Abstract

**Background:**

Administration of antineoplastic drugs may cause azoospermia driving to subfertility. Production of animal azoospermia models is essential for evaluating new treatment methods before therapeutic interventions in human setup. This study aimed to investigate the toxic effects of Busulfan (an anticancer drug) on some vital organs and describe the best method and appropriate dose of Busulfan to induce an animal azoospermia model.

**Methods:**

Rats were randomly assigned into four groups, treatment groups received 10 mg/kg, 40 mg/kg Busulfan intraperitoneally (IP), 5 mg/kg Busulfan intratesticular (IT), and control group. Blood, bone marrow, liver, renal, and testes samples were collected for histological (H&E staining), biochemical (serum levels of ALT, AST, ALP, creatinine, and urea), and hematological analyses.

**Results:**

Results revealed severe anemia and leukopenia in rats that received Busulfan via IP. By contrast, injection of 5 mg/kg Busulfan via IT did not cause anemia except with a mild decrease in RBC count. Non-significant differences in the M/E ratio were observed in all groups. The administration of 40 mg/kg of Busulfan led to evacuation and destruction in the spermatogenesis process with thin-walled seminiferous epithelium in most tubules, but in rats treated with 10 mg/kg of Busulfan, the normal spermatogenesis process was notified. IT injection of Busulfan contributed to the complete degradation of spermatogenesis in which all spermatogenic cells degenerated. In the renal tissue, hyperemia, extensive tubular necrosis degeneration, and hyaline casts were found after IP injection of Busulfan. In hepatic tissue, focal hemorrhagic, chronic cholangitis, and hepatocyte degeneration, and swelling were noticed. Biochemical analysis revealed apparent Busulfan toxicity of both hepatic and renal tissues in IP Busulfan-treated rats.

**Conclusions:**

In summary, we found that the intratesticular injection of low doses of Busulfan (5 mg/kg) is a relatively non-invasive and safe method for producing the rat azoospermia model causing the least toxicity on vital organs.

## Background

Infertility is a complex and multifactorial phenomenon with serious socioeconomic problems in developed nations. Based on statistics, infertility can affect about 8–12% of couples worldwide [1, 2]. Of note, male factor infertility is approximately 40–50% of all infertility cases [1]. It has been well documented that normal male fertility status depends on sperm production (spermatogenesis), transportation, and the proper collaboration of immune, endocrine, and neural systems [3, 4]. Spermatogenesis consists of a series of physiological, morphological, and biochemical changes in which lead to the polarization of progenitor cells toward mature sperms. This procedure can be disrupted after the occurrence of anomalies induced by congenital or genetic abnormalities, physical, chemical, and environmental factors, contributing to temporary or permanent infertility [3, 5, 6]. According to the latest report released by World Health Organization (WHO), the absence of spermatozoa in the ejaculate is known as azoospermia affects nearly about 1% of the male population and 10–20% of the infertile men [7, 8]. Regardless of genetic and congenital causes leading to azoospermia, chemotherapeutic agents can significantly alter the normal physiology of spermatogenesis, sperm parameters; halt the spermatogonial differentiation of progenitor cells, and deplete the germ cell pool [9].

Busulfan or 1, 4-butanediol dimethanesulfonate, used commonly for the treatment of most myeloproliferative syndromes, chronic myeloid leukemia (CML), lymphomas, and ovarian cancer, is a chemotherapeutic drug that can reduce proliferation rate by targeting the cells at the G1 phase of their growth process. This drug is capable of cross-linking between DNA-proteins or DNA-DNA, stoping cells at the mitosis/replication stage and causing apoptosis [10, 11]. Busulfan is also administrated in leukemia patients before bone marrow transplantation in combination with cyclophosphamide and clofarabine as a myelosuppressive/ myeloablative drug [12–16]. Noteworthy, both short- and long-term side effects have been reported on vital organs including the urinary bladder, liver, skin, gonads and, nervous system [17, 18]. An impaired spermatogenesis process can be found in cancer patients undergo Busulfan administration [19–21]. This study aimed to investigate the toxic effects of Busulfan on rat different organs such as the liver, kidneys, testes, and bone marrow using histological, biochemical, and cytological evaluations.

## Methods

### Experiment animals and ethics

Thirty-two male Wistar rats (6 to 8 weeks old with an average weight of 150 g) were obtained from the Animal Center of Tabriz University of Medical Sciences, Tabriz and housed in the standard conditions (22 ± 3 °C, 45–60% humidity) with unlimited access to water and chewing foods. This experiment was carried out in line with the guidelines of the Local Ethics Committee of Tabriz University of Medical Sciences (IR.TBZMED.VCR.REC.1397.333).

### Experimental design

After a week of acclimation, rats were randomly assigned into four groups (each in 8); (**I**): Control rats which did not receive any injections; (**II**): rats received a double dose of 10 mg/kg Busulfan (BUCELONTM 60, Celon Laboratories Ltd., Telangana state, India) intraperitoneally (IP) with 21 days of the interval; (**III**): rats received a single dose of 40 mg/kg Busulfan IP; and (**IV**): rats received a single dose of 5 mg/kg Busulfan intratesticular tissue (IT). To induce azoospermia, rats were sampled 56 days after the last injection [22–25].

### Tissue and blood sampling

To address the possible effect of Busulfan on cell blood count, blood samples were taken directly from the heart following deep anesthesia using 90 mg/kg Ketamine and 10 mg/kg Xylazine. Thereafter, rats were euthanized using an overdose of Ketamine and Xylazine. In this study, kidneys, liver, and testes were sampled, rinsed with phosphate-buffered saline to eliminate excess blood contaminations, and post-fixed in 4% paraformaldehyde (Merck).

### Cell blood count (CBC)

To calculate CBC, collected blood samples were analyzed manually. To this end, different parameters such as hematocrit (HCT), hemoglobin (Hb), red blood cells (RBCs), reticulocytes number (%) and total white blood cell count (WBC), and differential percent of neutrophils, lymphocytes, eosinophils, and monocytes were measured according to the previously published protocols [26].

### Evaluation of toxicity in bone marrow (BM)

BM aspiration and smears preparation were performed to evaluate the effects of Busulfan on Myeloid: Erythroid (M/E) ratio [27]. In short, the femurs were carefully isolated after euthanization. The extremities were cut using sterile scissors and marrow content was directly flushed out using PBS. Samples were centrifuged for 4 minutes at 3000 rpm. The pellets were washed twice with PBS and re-suspended in ice-cold physiologic buffered saline (PBS) containing 0.5% bovine serum albumin (BSA). The smear was prepared and stained with Giemsa solution [27].

### Biochemical evaluation

To examine the possible toxic effect of Busulfan on hepatic and renal tissues, serum levels of ALT (alanine aminotransferase), AST (aspartate aminotransferase), ALP (alkaline phosphatase), Creatinine, and Urea were evaluated. To collect the serum, blood samples were allowed to clot and centrifuged at 1500 g for 15 minutes and kept at − 80 °C for subsequent analyses.

### Histological evaluation

To evaluate the histopathological changes after Busulfan treatment, samples of liver, kidneys, and testes were embedded in paraffin and 5-μm thick sections were prepared using a microtome instrument. Subsequently, Hematoxylin and Eosin (H&E) staining was implemented and slides were monitored using light microscopy under different high power fields [28].

### Statistical analysis

Results of this experiment were analyzed to identify the significant levels using One-way ANOVA in GraphPad Prism software and presented as the mean ± SEM. *p* < 0.05 was considered statistically significant.

## Results

### Systemic injection of Busulfan led to rat mortality

**Fig. 1:**
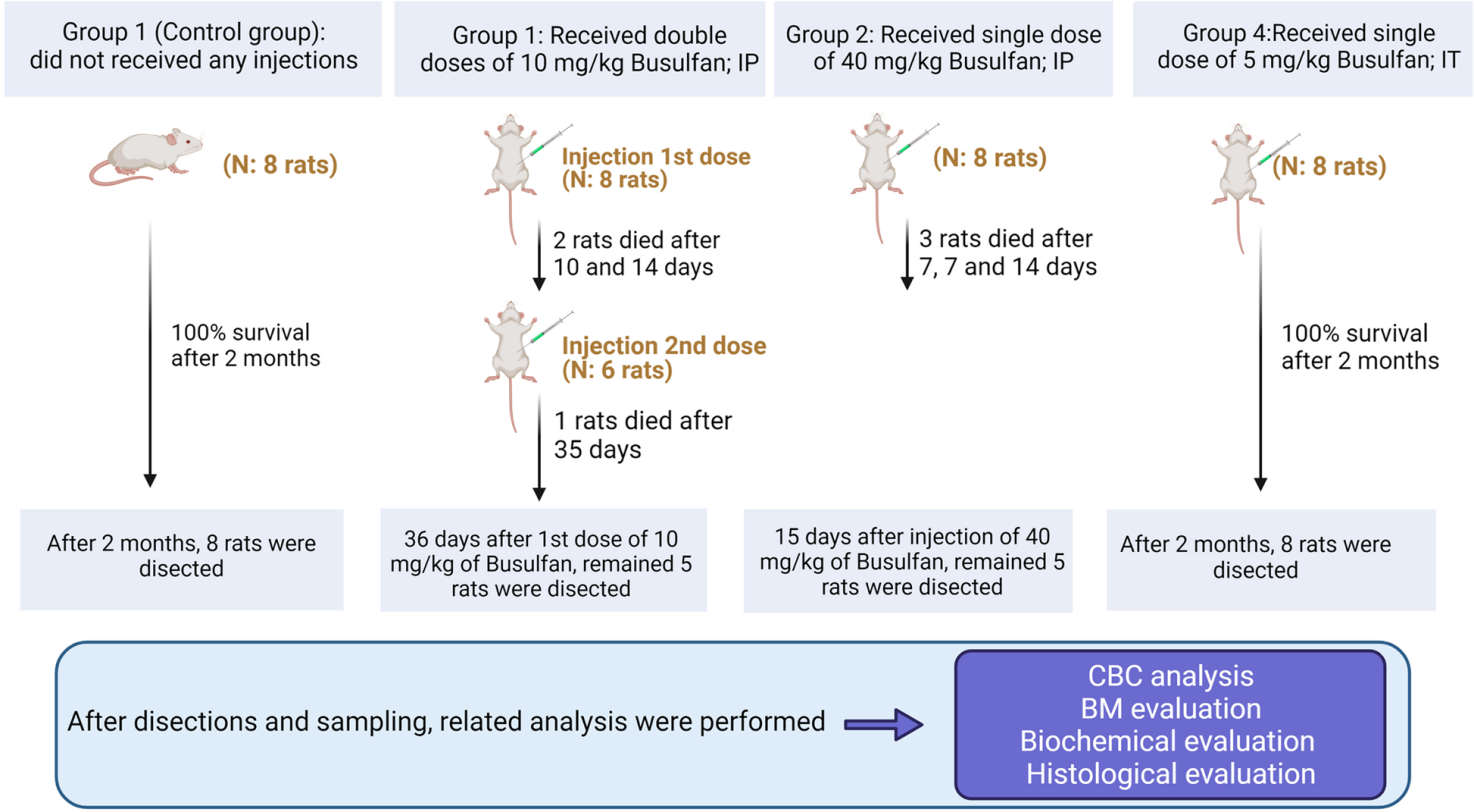
Graphical abstract as a concise, pictorial and visual summary of the grouping and methods of the article

Here, we monitored the mortality rate in rats received 10 (Group II) and 40 mg/kg (Group III) Busulfan. According to our data, the mortality rate reached 37.5% in both groups that received 10 and 40 mg/kg of Busulfan, showing dose-independent activity of Busulfan. By contrast, local injection of Busulfan did not yield mortality. Similarly, the control rats were survived until the end of the experiment. The general results are schematically presented in Fig.1.

### Busulfan can promote anemia and leukopenia in a dose-dependent manner

Data exhibited significant decreases in the hematological parameters such as mean RBC count, hematocrit, Hb, reticulocytes in rats received Busulfan via IP route (group II and III) compared to the control group (*p* < 0.05; Table 1). We found that anemia was more severe in the rats injected with 40 mg/kg of Busulfan compared to the other groups. Monitoring several parameters such as reticulocyte count, polychromasia, anisocytosis and the presence of basophilic stippling confirmed regenerative anemia in rats that received lower doses of Busulfan (10 mg/kg). By contrast, the type of anemia in rats treated with 40 mg/kg was non-regenerative and irreversible. These data showed dose-dependent activity and toxicity of Busulfan on hematological parameters. We noted the lack of significant differences in the levels of hematocrit, Hb, and reticulocytes between the control group and rats that received Busulfan IT. Of note, a mild decrease in RBC count was evident in the IT group. Along with these changes, 40 mg/kg Busulfan had the potential to suppress leukopoiesis indicated with neutropenia and lymphopenia as compared to other groups (*p* < 0.05). The levels of eosinophils and monocytes were not altered after injection of Busulfan compared to the control group (*p* > 0.05). According to our data, Busulfan did not change the M/E ratio in all groups (p > 0.05).

**Table 1:** The mean levels of complete blood count (CBC) in different studied groups

Parameters	Groups			
	I	II	III	IV
**PCV (%)**	42 ± 1.36^a^	21.8 ± 1.62^b^	26 ± 0.31^c^	42.4 ± 0.6^a^
**Hgb (g/dl)**	13.6 ± 0.45^a^	7.8 ± 0.55^b^	8.75 ± 0.38^b^	13.98 ± 0.28^a^
**RBC (×10**^**6**^**/μL)**	7.85 ± 0.31^a^	3.41 ± 0.13^b^	4.08 ± 0.17^b^	6.4 ± 0.43^c^
**Reticulocytes (%)**	2.36 ± 0.52^a^	6.5 ± 0.99^b^	0.8 ± 0.05^c^	2.14 ± 0.39^a^
**WBC (× 10**^**3**^**/**μL**)**	10.80 ± 0.47^a^	4.33 ± 0.2^b^	2.24 ± 0.26^c^	6.18 ± 0.22^d^
**Lymphocytes (× 10**^**3**^**/μL)**	7.14 ± 0.51^a^	4.49 ± 0.23^b^	2.09 ± 0.31^c^	4.6 ± 0.27^b^
**Neutrophils (× 10**^**3**^**/μL)**	3.5 ± 0.15^a^	0.33 ± 0.01^b^	0.22 ± 0.06^b^	1.03 ± 0.12^c^
**Monocytes (× 10**^**3**^**/μL)**	0.19 ± 0.06^ac^	0.1 ± 0.01^a^	0.03 ± 0.008^ab^	0.28 ± 0.07^c^
**Eosinophils (× 10**^**3**^**/μL)**	0.02 ± 0.03^ab^	0.01 ± 0.009^a^	0.01 ± 0.007^a^	0.18 ± 0.05^b^
**M/E**	1.76 ± 0.25^a^	1.38 ± 0.09^a^	1.78 ± 0.34^a^	1.39 ± 0.16^a^

Data are presented as means ± SEM. I, negative control group (*n* = 8); II, intraperitoneal (IP) 10 mg/kg Busulfan injection group (*n* = 5); III, intraperitoneal (IP) 40 mg/kg busulfan injection group (*n* = 5); IV, intratesticular (IT) 5 mg/kg/side Busulfan injection group (*n* = 8); *PCV* packed cell volume, *Hgb* Haemoglubin, *RBC* Red blood cells, *M/E* Myeloid/erythroid ratio. Different letters (a, b, and c) within a column denote statistically significant differences between groups

### Liver and kidneys biomarkers were altered after Busulfan injection

Based on the biochemical analysis (Table 2), IP injection of 10 and 40 mg/kg Busulfan decreased significantly creatinine levels compared to the control group (*p* < 0.05). Data showed a slight increase in the serum level of urea in Busulfan-injected groups, however, these values did not reach statistically significant levels (*p* > 0.05). We found that ALT levels were significantly decreased in the 40 mg/kg-received IP group compared to other groups (*p* < 0.05; Table 2). Non-significant differences were notified between group IV (IT injected Busulfan) and control rats (p > 0.05) in terms of ALT. According to our data, 10 mg/kg Busulfan increased AST in comparison to IT-injected rats (*p* < 0.05). Surprisingly, 10 and 40 mg/kg Busulfan decreased ALP significantly compared to control and IT groups (p < 0.05). Data showed that local injection of Busulfan did not alter ALT, AST, ALP, creatinine, and urea. The evident reduction of hepatic enzymes in IP-injected rats possibly correlates with prominent hepatic toxicity. These features coincided with slight to mild alteration in renal function.

**Table 2:** The mean levels of some biochemical parameters in different studied groups

Parameters	Groups			
	I	II	III	IV
**Creatinine (mg/dL)**	0.76 ± 0.02^a^	0.62 ± 0.02^b^	0.62 ± 0.01^b^	0.84 ± 0.07^a^
**Urea (mg/dL)**	52.33 ± 0.32^a^	55.26 ± 2.12^a^	58.38 ± 3.92^a^	58 ± 1.5^a^
**ALT (U/L)**	63.12 ± 1.51^a^	61.2 ± 4.07^ab^	50 ± 1.14^b^	65.7 ± 6.03^a^
**AST (U/L)**	194.8 ± 9.5^ab^	260.6 ± 15.11^a^	193.3 ± 42.92^ab^	170.2 ± 24.12^b^
**ALP (U/L)**	563.6 ± 21.09^a^	508.4 ± 25.19^b^	296.8 ± 12.43^c^	516.4 ± 28.33^ab^

### Histopathology results revealed defective consequences of Busulfan

**Fig. 2:**
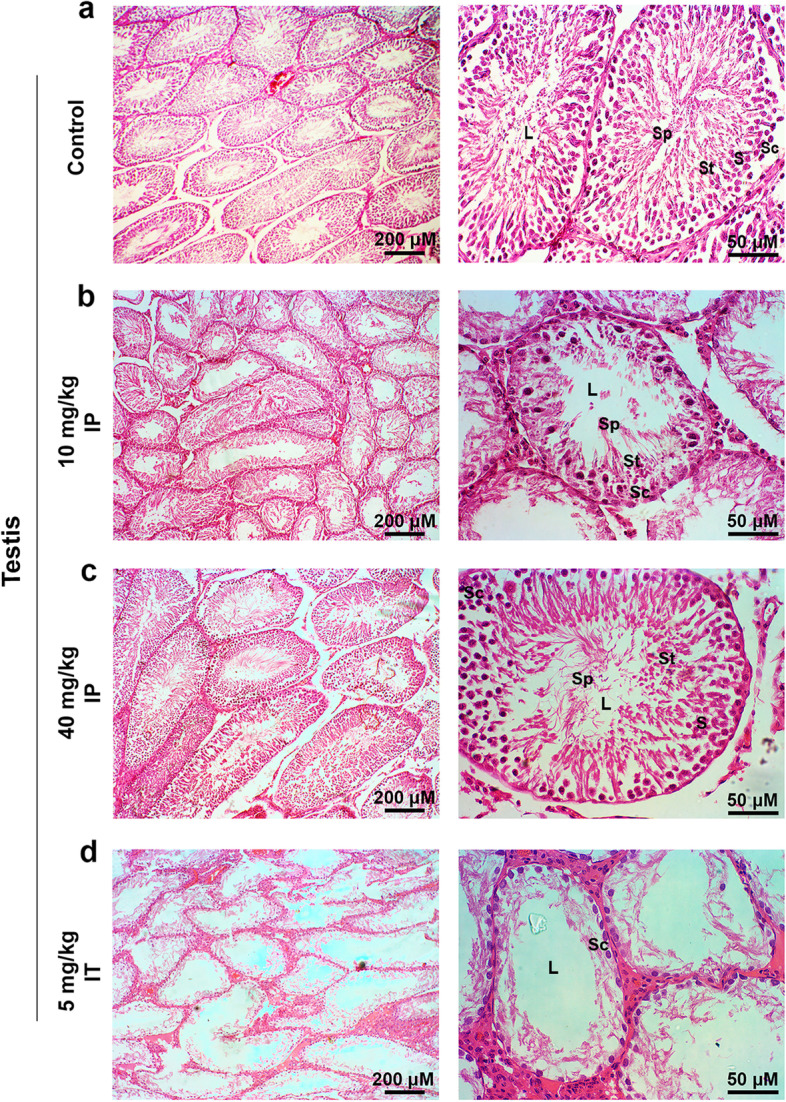
H & E-stained testicular sections of Busulfan-injected rats: (**a**) normal spermatogenesis was observed in histological evaluation of testicular tissue sections of the control group with thick-walled seminiferous tubules along with multiple layers of spermatogenesis cells and elongated spermatozoa within or close to the luminal surface. (**b**) Testicular sections of group II (received 10 mg/kg of Busulfan IP) showed minor evacuation and destruction in the spermatogenesis process with multiple vacuoles, thin-walled seminiferous epithelium; however, spermatozoa were present in the lumen and quite notable numbers of spermatogonial cells in the innermost layer of the seminiferous tubules. (**c**) Testicular tissue sections of the third group (received 40 mg/kg of Busulfan IP) revealed the normal spermatogenesis process with the presence of spermatogenic cells and elongated spermatozoa seminiferous tubules and no destruction was observed in the tubules. (**d**) Histological evaluations demonstrated that most of the seminiferous tubules and spermatogenesis cells of testicular tissue are destroyed without any spermatozoa in the lumen and the seminiferous epithelium became thinner with only one layer of germ cells remaining at the periphery of tubules 60 days after a single injection (IT) of 5 mg/kg/rat Busulfan. Images scale bar; Left, 200 μm and right, 50 μm. Abbreviations: L, lumen; Sc, spermatogonia; S, spermatocytes; St, spermatid and Sp, spermatozoa, (*n* = 8)

Histological evaluation of testicular tissue in normal rats revealed the thick-walled seminiferous tubules with multiple layers of spermatogenesis cells and elongated spermatozoa within or close to the luminal surface in the control group (Fig. 2a). According to our data, in rats that received 10 mg/kg of Busulfan via IP minor evacuation and destruction in the spermatogenesis process were notified. Multiple vacuoles, the thin-walled seminiferous epithelium was observed without toxic effects on spermatogenesis in which quite notable numbers of spermatogonial cells in the innermost layer of the seminiferous tubules were observed (Fig. 2b). In the 40 mg/kg Busulfan group, normal spermatogenesis process along with the presence of spermatogenic cells in seminiferous tubules and elongated spermatozoa in the luminal surface of seminiferous tubules were obtained (Fig. 2c). Despite these results, complete evacuation of testicular sections, degradation of spermatogenesis, and severe tubular disorganization were seen in in rats that received 5 mg/kg of Busulfan via IT. Except for the number of germ cells, all spermatogenic cells were reduced indicated with azoospermia (Fig. 2d).

**Fig. 3:**
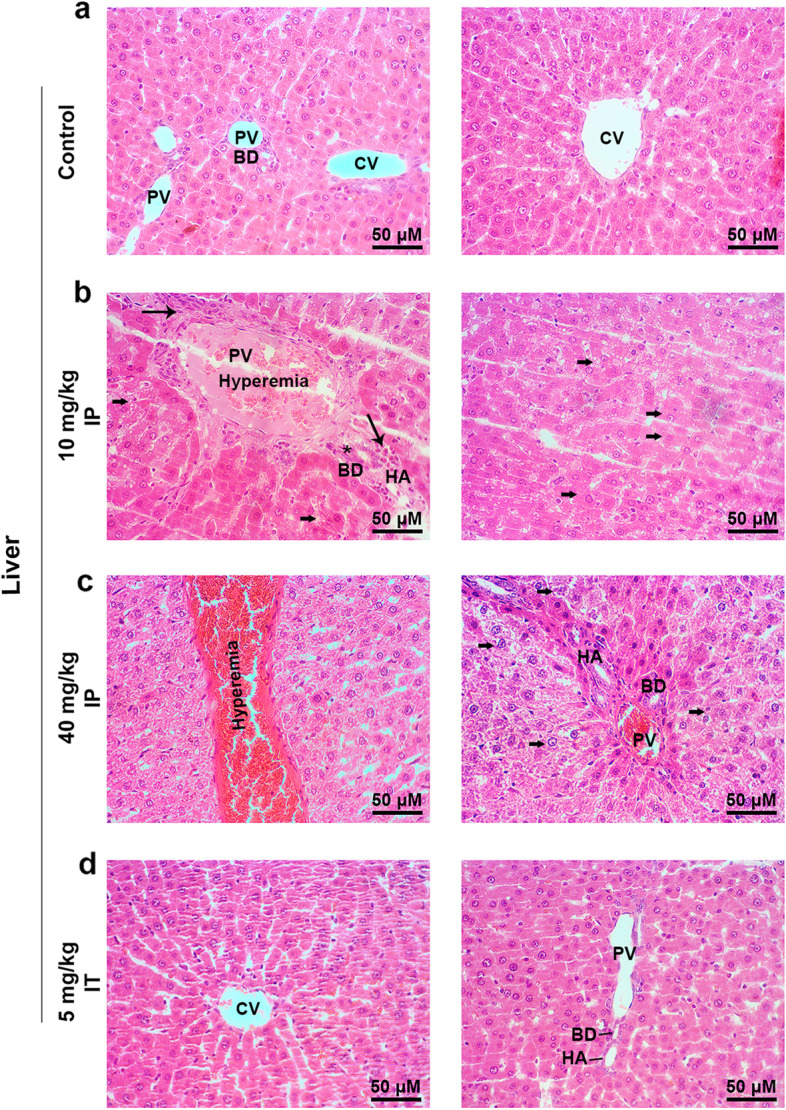
H & E staining of hepatic sections after Busulfan injection: (**a**, **d**) No adverse effects were seen on hepatic tissue after injection of 5 mg/kg Busulfan via IT compared to the control rats. (**b**, **c**) Following IP injections of Busulfan, numerous focal hemorrhagic, mild cholangitis, hyperemia, swelling, and hepatocytes degeneration and infiltrated inflammatory cells around portal tracts were seen in the liver tissue sections. Our findings showed notable hepatic toxicity in rats that received 40 mg/kg IP Busulfan which was quite severe than 10 mg/kg rats. Images scale bar; 50 μm. Abbreviations: CV, central vein; PV, portal vein; BD, bile duct; HA, hepatic artery; thin arrows, infiltrated inflammatory cells; thick arrows, swelled and degenerated hepatocytes; Star, mild cholangitis, (*n* = 8)

**Fig. 4:**
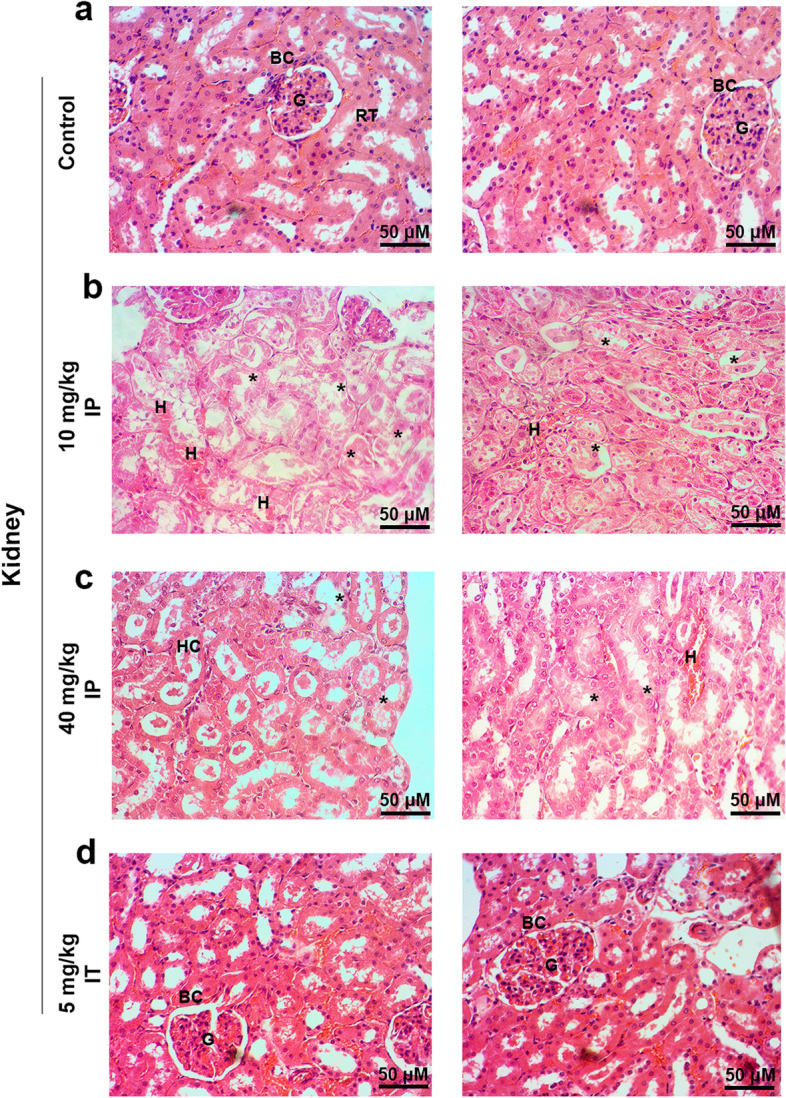
H & E staining of renal sections after Busulfan injection: (**a**, **d**) No adverse were seen effects on renal tissue after injection of 5 mg/kg of Busulfan via IT compared to the control rats (**b**, **c**) Following IP injections of Busulfan, focal hyperemia, hyaline casts, extensive renal tubules necrosis, swelling and degeneration without glomerulitis were seen in the renal tissue sections. Our findings showed notable renal toxicity in rats that received 40 mg/kg IP Busulfan which was quite severe than 10 mg/kg rats. Images scale bar; 50 μm. Abbreviations: BC, bowman capsule; G, glomerulus; RT, renal tubules; H, hyperemia; HC, hyaline casts; Star, swelled, degenerated, and necrotic renal tubular epithelial cells, (*n* = 8)

To evaluate the possible toxicity of Busulfan in other tissues, kidneys, and liver tissue were also monitored using the histological examination. In the hepatic tissue, IP injection of Busulfan, either 10 or 40 mg/kg, exhibited numerous focal hemorrhagic, mild cholangitis, hyperemia, swelled hepatocytes with infiltrated inflammatory cells around portal tracts, indicating moderate inflammation after the injection of Busulfan (Fig. 3). In kidneys, focal hyperemia, hyaline casts, extensive renal tubules necrosis, swelling, and degeneration without glomerulitis in IP Busulfan-treated rats (Fig. 4). Our findings showed notable renal and hepatic toxicity in rats that received 40 mg/kg IP Busulfan which was more than that of 10 mg/kg rats. In contrast, a low dose of Busulfan (5 mg/kg) injected IT showed no adverse effects on both hepatic and renal tissue resembling the control rats.

## Discussion

Until the present time, attempts have been made to deal with the side effects of gonadotoxic agents in both genders to restore the reproduction capacity. It has been shown that chemotherapy could induce temporary non-obstructive azoospermia, oligospermia, and in severe cases permanent infertility, depending on the amount and duration of exposure to the chemotherapy agents [4, 29]. Clinically, chemotherapy drugs promote progenitor cell injury and reduce spermatogenesis, leading to short- or long-term infertility [4, 29, 30]. Of these compounds, Busulfan as an anticancer drug is a common agent used for the induction of azoospermia in animal models [17, 24, 31, 32].

To be specific, different doses of Busulfan [10–50 mg/kg, IP and 4–6 mg/kg, IT] have been used to induce azoospermia in animal species [32–34]. Commensurate with these descriptions, it seems that finding appropriate dosage can help us to establish a more efficient method to achieve azoospermia model in animals with the least side effects. Therefore, the current investigation was designed to examine the most effective dose of Busulfan for azoospermia induction and evaluate the possible toxic effect on the liver and kidneys in both types of administration via IP and IT.

Our finding revealed that an ideal azoospermia model was successfully developed in rats that received 5 mg/kg of Busulfan via IT. In these rats, relatively higher levels of epithelial cells were depleted within the seminiferous tubules coincided with the lack of mature spermatozoa inside the lumen. Additionally, thin-walled seminiferous tubules with a single-layer furnishing cell were evident. Despite these findings, no toxic effects were found in the kidneys and liver. It has been shown Busulfan promotes toxic effects on tissues via the irreversible alkylation of biomolecules inside the cells [24, 35]. IP injection of Busulfan (10 mg/kg) led to a reduced spermatogenesis process and in some tubules, spermatozoa could be seen within the lumens. These effects were more evident in rats that received 40 mg/kg Busulfan compared to the group treated with 10 mg/kg Busulfan. These data confirmed that local administration of Busulfan can accelerate degeneration procedure inside the rat testes even in lower doses compared to the higher doses administrated via IP.

Despite the severe testicular tissue degeneration via IT injection, the levels of hepatic and renal tissue biomarkers and hematological parameters were relatively intact compared to the normal condition. By contrast, severe non-regenerative anemia, moderate to severe lymphopenia, and neutropenia with the reduction of ALT, ALP, and creatinine were found in rats that received 40 mg/kg Busulfan and these effects were less in the group treated with 10 mg/kg Busulfan via IP. No systemic effects were found in rats that had undergone IT Busulfan injection. The least toxic effects of Busulfan in the IT group could be associated with the low circulation of Busulfan in the bloodstream while in the IP injection Busulfan can bio-distribute faster to the remote sites especially kidneys and liver [33, 36–38]. The most probable cause of mortality in animal modeling following Busulfan admiration is associated with the suppression of leukopoiesis and hematopoiesis. In this regard, 50 mg/kg Busulfan has been reported as a lethal dose due to its cytotoxic effects on the hematopoietic system [39]. In a study conducted by Wang and colleagues, the survival rate of mice receiving Busulfan at a dose of 10 and 40 mg/kg was 0 and 13.3%, respectively [39]. Depending on the dose, injection site, and duration of exposure to Busulfan, the degree of damage to seminiferous tubules will also vary [22, 34, 40]. Xie and co-workers, used different doses of Busulfan (20, 30, 40 mg/kg) via IP in the mouse model. They showed a significant reduction in the number of germ cells in the seminiferous tubules at a dose of 40 mg/kg compared to lower doses. After injecting a high dose of Busulfan, all germ cells were destroyed over time [34]. Considering the least and transient destructive effects for Busulfan in lower doses, long-term exposure to higher doses of Busulfan could exert more devastating effects on germ cells, resulting in reduced regeneration within the seminiferous tubules leading to permanent infertility [39]. Even in higher doses, seminiferous tubules were not completely depleted from germ cells [32, 41]. Consistent with our data, Anjamrooz and colleagues found a decline in epididymis sperm count in mice following the injection of 20–50 mg/kg Busulfan. Like our data, no abnormalities in sperm parameters were found in the mice that received 10 mg/kg Busulfan. Taken together, our results clearly illustrate that the IT injection of the low dose of Busulfan (5 mg/kg) is the safest method for induction of azoospermia in rats without toxicity in other organs.

## Conclusion

We used different doses of Busulfan for the development of azoospermia in the rat model. To this end, 5 mg/kg Busulfan was injected via IT. This dosage and route of administration induced prominent azoospermia without hepatotoxicity and renal damage. We showed that IP injection of Busulfan (10 and 40 mg/kg) can lead to liver and kidneys injury without sufficient azoospermia induction. Further investigations are needed to increase knowledge regarding the non-toxic and effective dose of Busulfan on different species.

## Data Availability

All data analyzed in this study is available from the corresponding author on reasonable request.

## Abbreviations

WHO: World health organization
CML: Chronic myeloid leukemia
IP: Intraperitoneally
IT: Intratesticular tissue
CBC: Cell blood count
HCT: Hematocrit
Hb: Hemoglobin
RBCs: Red blood cells
WBC: White blood cell
BM: Bone marrow
M/E: Myeloid: Erythroid
PBS: Physiologic buffered saline
BSA: Bovine serum albumin
ALT: Aalanine aminotransferase
AST: Aspartate aminotransferase
ALP: Alkaline Phosphatase
H&E: Hematoxylin and Eosin
